# Valproic acid restricts mast cell activation by *Listeria monocytogenes*

**DOI:** 10.1038/s41598-022-20054-5

**Published:** 2022-09-20

**Authors:** Rodolfo Soria-Castro, Yatsiri G. Meneses-Preza, Gloria M. Rodríguez-López, Alfredo Ibarra-Sánchez, Claudia González-Espinosa, Sonia M. Pérez-Tapia, Fabián Flores-Borja, Sergio Estrada-Parra, Alma D. Chávez-Blanco, Rommel Chacón-Salinas

**Affiliations:** 1 Departamento de Inmunología, Escuela Nacional de Ciencias Biológicas, Instituto Politécnico Nacional (ENCB-IPN), Carpio Y Plan de Ayala S/N Col. Santo Tomás, C.P. 11340 Mexico City, Mexico; 2 Departamento de Microbiología e Inmunología, Facultad de Medicina Veterinaria y Zootecnia, Universidad Nacional Autónoma de México, Mexico City, Mexico; 3 Departamento de Farmacobiología, Centro de Investigación y de Estudios Avanzados (Cinvestav), Unidad Sede Sur, Mexico City, Mexico; 4 Unidad de Desarrollo e Investigación en Bioprocesos (UDIBI), Escuela Nacional de Ciencias Biológicas, Instituto Politécnico Nacional (ENCB-IPN), Mexico City, Mexico; 5 Centre for Oral Immunobiology and Regenerative Medicine, Barts & The London School of Medicine and Dentistry, Queen Mary University of London, London, UK; 6 Subdirección de Investigación Básica, Instituto Nacional de Cancerología (INCan), Av. San Fernando No. 22. Col. Sección XVI, C.P. 14080 México City, México

**Keywords:** Mast cells, Immunology, Innate immune cells

## Abstract

Mast cells (MC) play a central role in the early containment of bacterial infections, such as that caused by *Listeria monocytogenes* (L.m). The mechanisms of MC activation induced by L.m infection are well known, so it is possible to evaluate whether they are susceptible to targeting and modulation by different drugs. Recent evidence indicates that valproic acid (VPA) inhibits the immune response which favors L.m pathogenesis in vivo. Herein, we examined the immunomodulatory effect of VPA on L.m-mediated MC activation. To this end, bone marrow-derived mast cells (BMMC) were pre-incubated with VPA and then stimulated with L.m. We found that VPA reduced MC degranulation and cytokine release induced by L.m. MC activation during L.m infection relies on Toll-Like Receptor 2 (TLR2) engagement, however VPA treatment did not affect MC TLR2 cell surface expression. Moreover, VPA was able to decrease MC activation by the classic TLR2 ligands, peptidoglycan and lipopeptide Pam3CSK4. VPA also reduced cytokine production in response to Listeriolysin O (LLO), which activates MC by a TLR2-independent mechanism. In addition, VPA decreased the activation of critical events on MC signaling cascades, such as the increase on intracellular Ca^2+^ and phosphorylation of p38, ERK1/2 and -p65 subunit of NF-κB. Altogether, our data demonstrate that VPA affects key cell signaling events that regulate MC activation following L.m infection. These results indicate that VPA can modulate the functional activity of different immune cells that participate in the control of L.m infection.

## Introduction

Mast cell (MC) progenitors originate in the bone borrow and migrate from the blood to peripheral tissues like the skin, the connective tissue, and the mucosal epithelia, which are usually the first sites of invasion by several pathogens. MC are considered sentinel cells of the innate immune system and one of the first responders to infection^1^. MC are typically associated with the development of type I hypersensitivity reactions^2^, but mounting evidence indicates a critical role triggering the host immune response to infection^3^. MC express Pattern Recognition Receptors (PRRs) which mediate the release of different preformed and de novo synthesized inflammatory mediators^4^. Thus, MC participate in the host response to pathogens such as viruses^5^, fungi^6^, parasites^7^, and bacteria^8^, including *Listeria monocytogenes* (L.m)^9^.

L.m is a Gram-positive, facultative intracellular bacterium that induces gastrointestinal disease in immunocompetent individuals, meningitis in immunocompromised individuals and abortion in pregnant women^10^. Therefore, the immunological status of the host is crucial for the control of infection by this pathogen^11^.

Cells of the innate immune system including macrophages, dendritic cells, neutrophils, NK cells, and MC are involved in the early control of L.m infection^11^. In vitro and in vivo models of listeriosis have shown that MC play a critical role controlling L.m infection by degranulating and releasing pro-inflammatory cytokines which induce early recruitment of neutrophils to the site of infection^9,12^. In addition, MC restrict L.m growth through endocytosis, reactive oxygen species (ROS) production^13,14^, and by the release of extracellular traps^15^. Since the mechanisms of MC activation in response to L.m are well known^13–16^, it is possible to evaluate whether they are susceptible to modulation by different drugs. These analyses could elucidate new molecular targets for these molecules and contribute to our understanding of the pharmacological control of innate immunity responses orchestrated by MC.

Valproic acid (VPA) is a short chain fatty acid that is used as the first-line drug for the control of different forms of epilepsy and in the treatment of other neurological disorders^17^. Several clinical trials showed that VPA has a potential anticancer effect, due to its ability to inhibit histone deacetylases (HDAC) and thereby reverse some epigenetically silenced genes in tumor cells^18^. Moreover, VPA has shown inhibitory effects on key cell signaling pathways involved in the activation of different immune cells^19^.

The immunosuppressive effect of VPA seems to favor the infection of different pathogens by altering the adequate deployment of the immune response^20^. For instance, patients with bipolar disorder undergoing VPA treatment show an increased risk of respiratory infections^21^. Moreover, VPA affects the immune response of mice infected with *Klebsiella pneumoniae*, *Candida albicans*^22^ and L.m^23^, either by modulating the response of phagocytic cells to Pathogen-Associated Molecular Patterns (PAMPs)^22,24^ or by interfering with the activation of the cell signaling cascade required for IFN-γ production by NK cells^23^. However, whether VPA affects MC response during infectious processes is unknown. Therefore, in this study, we examined the ability of VPA to modulate MC activation by L.m.

## Results

### Valproic acid impairs *Listeria monocytogenes*-mediated mast cell activation

**Figure 1:**
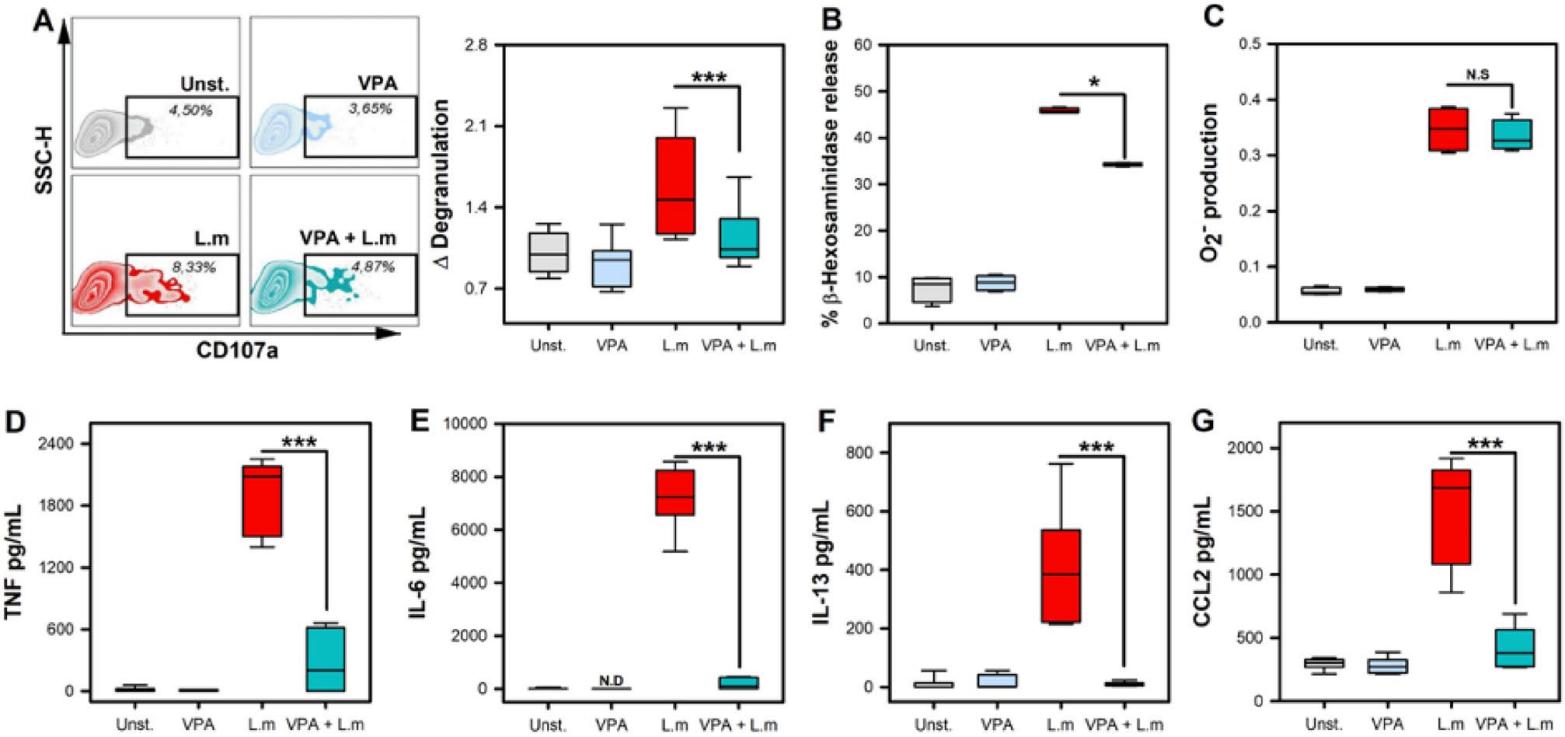
Valproic acid impairs mast cell degranulation and cytokine production in response to *Listeria monocytogenes* infection. (**A**) Bone marrow-derived mast cells (BMMC) were pre-incubated with 2 mM VPA for 18 h and then stimulated with *Listeria monocytogenes* (L.m) for 90 min. Degranulation was determined by flow cytometry. The left panel shows representative Zebra-plots. The right panel shows the fold change in the percentage of FcεRI + /CD107a + cells with respect to the average value of unstimulated BMMC. (n = 9 per group; ***p < 0.001; Kruskal–Wallis test). (**B**) BMMC were pre-incubated with 2 mM VPA for 18 h and then stimulated with L.m for 90 min. The MC degranulation percentage was determined by the β-hexosaminidase release assay. (n = 4 per group; *p < 0.05; Kruskal–Wallis test). (**C**) BMMC were pre-incubated with 2 mM VPA for 18 h and then stimulated with L.m for 2 h. The O_2_^-^ evaluation was performed by the NBT reduction assay. The panel shows the fold change in the absorbance with respect to unstimulated BMMC. (n = 4 per group; N.S = Not Significance; Kruskal–Wallis test). (**D-G**) BMMC were pre-incubated with 2 mM VPA for 18 h and then stimulated with L.m for 24 h. Cytokines levels were evaluated in culture supernatants by ELISA. (**D**) TNF, (**E**) IL-6, (**F**) IL-13, (**G**) CCL2. (n = 6 per group; N.D = Not Detected; ***p < 0.001; Kruskal–Wallis test).

VPA restricts the activation of cells that participate in the immune response and allergic diseases, including FcεRI-dependent triggering of MC^25^. However, it is unknown whether VPA alters MC activation during bacterial infection. To this end, we first evaluated the effect of 2 mM VPA on L.m-induced MC degranulation. VPA dose was selected based on previous studies showing that this dose was not toxic for BMMC^25^ and it is within the range detected in sera of patients with bipolar disorders treated with this drug^26^. VPA treatment of BMMC obtained from female mice (f-BMMC) induced a significant reduction in the percentage of degranulated cells (CD107a +) after 90 min of stimulation with L.m (*p* < 0.001) (Fig. 1A), and in the amount of β-hexosaminidase released (*p* < 0.05) (Fig. 1B).

Because ROS generation is another hallmark of MC activation by distinct stimuli^15^, we evaluated whether VPA affected superoxide anion (O_2_^−^) production. Following 2 mM VPA treatment and stimulation for 30 min with L.m, we noticed that VPA, did not affect L.m-induced O_2_^−^ level (Fig. 1C).

MC activation by L.m also leads to the release of different pro-inflammatory cytokines^13^. Hence, we investigated whether 2 mM VPA could affect cytokine production by MC infected with L.m. We observed that VPA decreased the release of TNF, IL-6, IL-13, and CCL2 (*p* < 0.001) during L.m infection of BMMC derived from female mice (Fig. 1D-G). As previous studies have demonstrated that MC have a sexual dimorphic response^27^, we evaluated whether male mice-derived MC (m-BMMC) could respond different to VPA in the context of L.m infection. After expansion, m-BMMC cultures showed a similar purity to f-BMMC (Fig. S1A). Moreover, m-BMMC showed a diminished IL-6 production in response to L.m when compared to f-BMMC (*p* < 0.05) (Fig. S1B). However, m-BMMC IL-6 release induced by L.m was also attenuated in the same magnitude by VPA (*p* < 0.01) (Fig. S1C), as was observed in f-BMMC. Altogether, these results indicate that VPA selectively affects some mechanisms of MC activation induced by L.m infection.

### Valproic acid affects TLR2 ligand-mediated mast cell activation without compromising TLR2 expression

**Figure 2:**
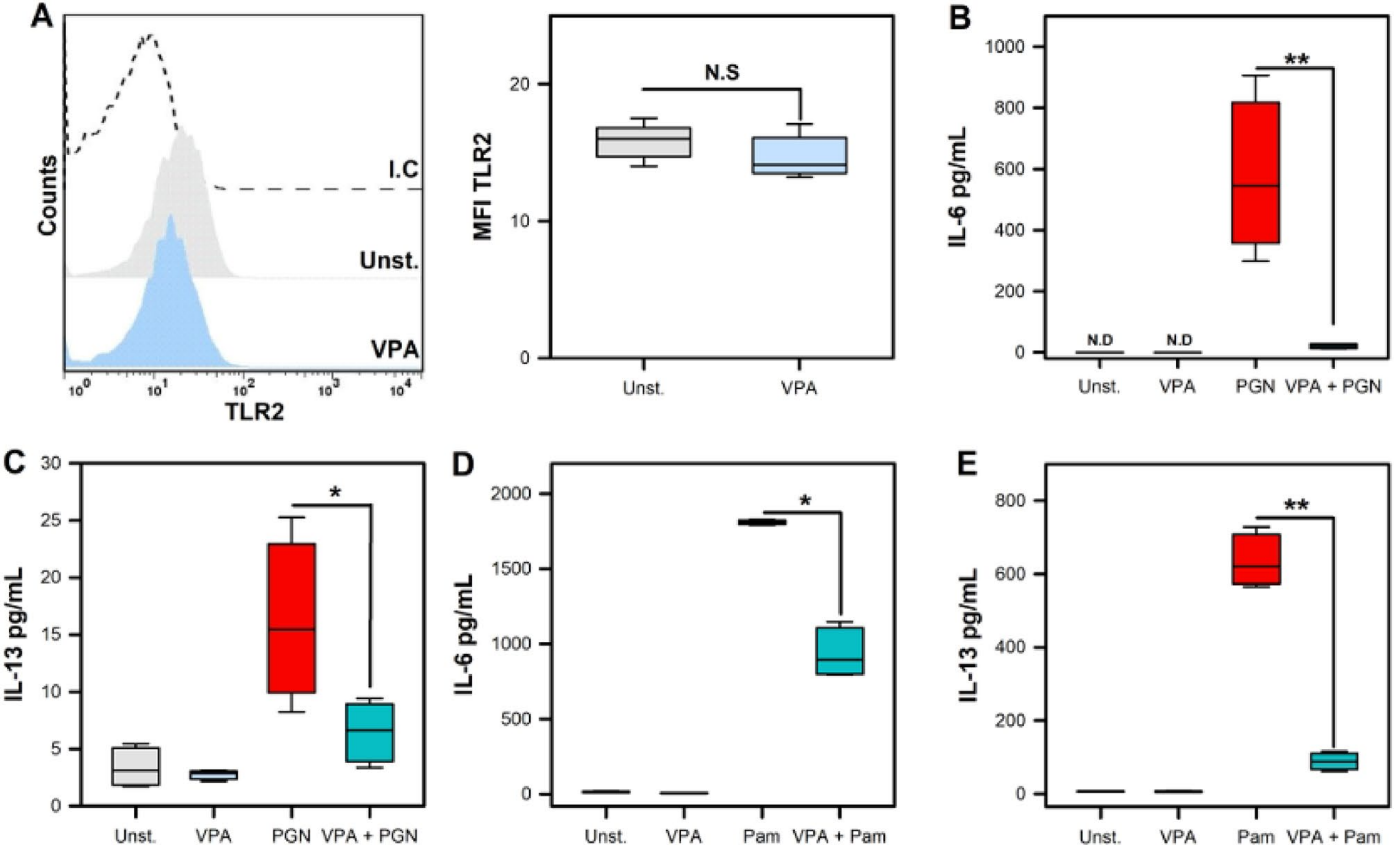
Valproic acid affects mast cell activation by TLR2 ligands. (**A**) BMMC were pre-incubated with 2 mM VPA for 18 h. Then, the cells were marked with anti-TLR2-biotin or biotinylated isotype control (I.C) and stained with streptavidin-APC. Staining was evaluated by flow cytometry. The left panel shows representative histograms for surface TLR2 expression. The right panel shows the median fluorescence intensity (MFI) with respect to unstimulated BMMC. (n = 4 per group; N.S = Not Significance; Mann–Whitney test). (**B**,**C**) BMMC were pre-incubated with 2 mM VPA for 18 h and then stimulated with *S. aureus* Peptidoglycan (PGN). Cytokine levels were evaluated in culture supernatants by ELISA. (**B**) IL-6 and **(C)** IL-13. (**D**-**E**) BMMC were pre-incubated with 2 mM VPA for 18 h and then stimulated with Pam3CSK4 (Pam) for 24 h. (**D**) IL-6 and (**E**) IL-13. (n = 4 per group; *p < 0.05, **p < 0.01; Kruskal–Wallis test).

Toll-Like Receptor (TLR2) plays an important role in innate immune defense against L.m and the early secretion of pro-inflammatory cytokines^28^. The importance of MC TLR2 during infection with *Francisella tularensis*^29^ and L.m^14^ has been well established. Therefore, we evaluated whether VPA affected TLR2 expression. Thereby, BMMC were treated with 2 mM VPA for 18 h and then TLR2 MC surface expression was assessed by flow cytometry. We did not find that VPA significantly decreased TLR2 expression in BMMC (Fig. 2A). Next, we assessed whether the MC response to TLR2 agonists was affected by VPA. We found that the pretreatment of BMMC with VPA had a profound effect on IL-6 and IL-13 production in response to the classic TLR2 ligands, *Staphylococcus aureus* Peptidoglycan (PGN) and the synthetic ligand Pam3CSK4 (Pam) (*p* < 0.05; *p* < 0.01) (Fig. 2B-E). These results indicate that VPA alters MC TLR2 activation and could be implicated in the downmodulated response of MC to L.m.

### Valproic acid affects Listeriolysin O-induced by mast cell activation

**Figure 3:**
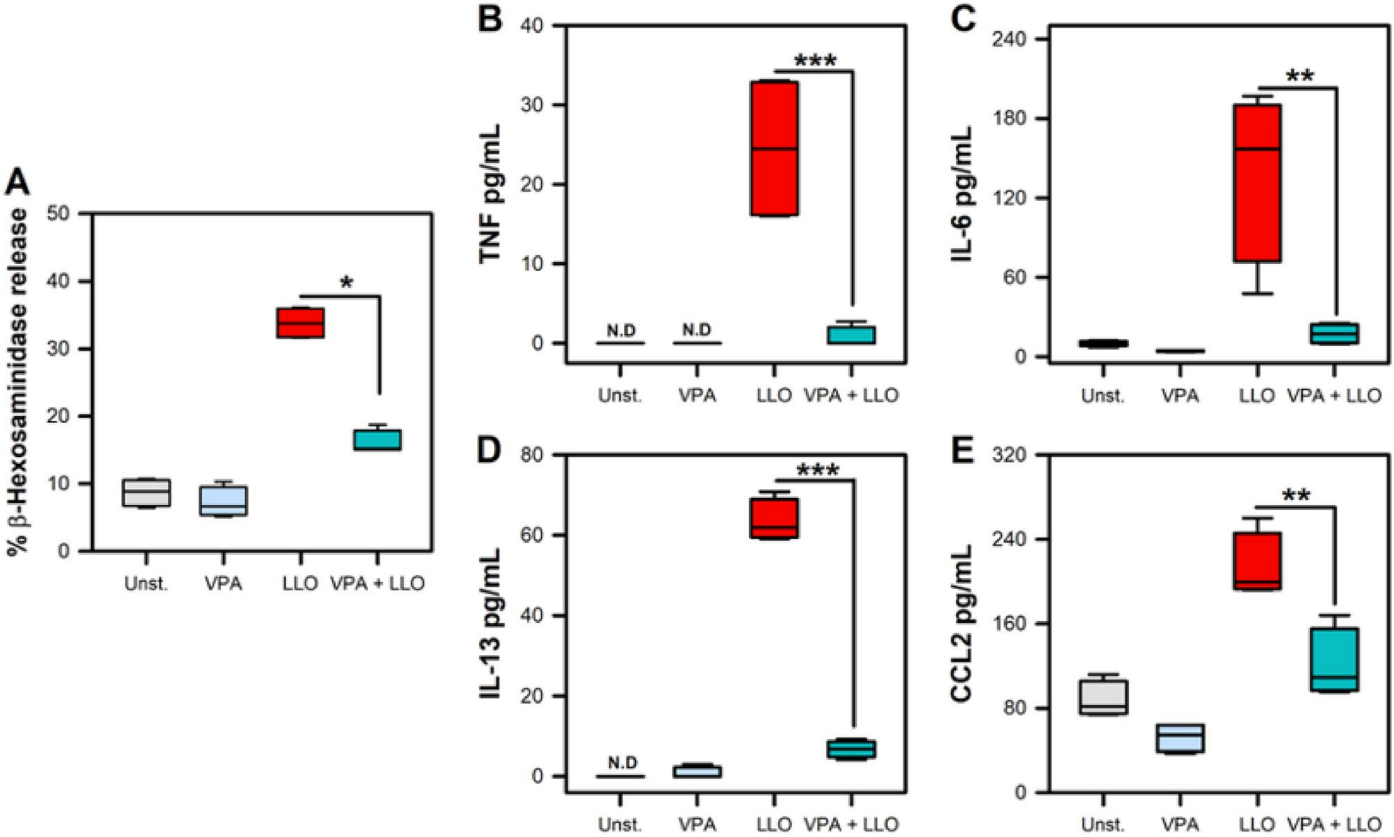
Valproic acid reduces mast cell cytokine production in response to Listeriolysin O. (**A**) BMMC were pre-incubated with 2 mM VPA for 18 h and then stimulated with Listeriolysin O (LLO) for 90 min. The MC degranulation percentage was determined by the β-hexosaminidase release assay. (n = 4 per group; *p < 0.05; Kruskal–Wallis test). (**B**-**E**) BMMC were pre-incubated with 2 mM VPA for 18 h and then stimulated with Listeriolysin O (LLO) for 24 h. Cytokines levels were evaluated in culture supernatants by ELISA. (**B**) TNF, **(C)** IL-6, (**D**) IL-13**,** (**E**) CCL2. (n = 4 per group; N.D = Not Detected; **p < 0.01, ***p < 0.001; Kruskal–Wallis test).

L.m also activates MC in a TLR2-independent manner through Listeriolysin O (LLO) toxin which activates MC in a cholesterol dependent pathway^14,30^. Therefore, we evaluated whether VPA could affect LLO-induced degranulation and cytokine secretion by MC. We found that BMMC treated with 2 mM VPA and stimulated with LLO showed a significant decrease in MC degranulation, as reflected by β-hexosaminidase release (*p* < 0.05) (Fig. 3A), as well as a decreased production of TNF, IL-6, IL-13, and CCL2 when compared with LLO-stimulated BMMC (*p* < 0.01; *p* < 0.001) (Fig. 3B-E). Therefore, these results show that VPA also affected MC activation independent of TLR2 through L.m. LLO.

### Valproic acid decreases the activation of key cell signaling pathways in *Listeria monocytogenes*-activated mast cells

**Figure 4:**
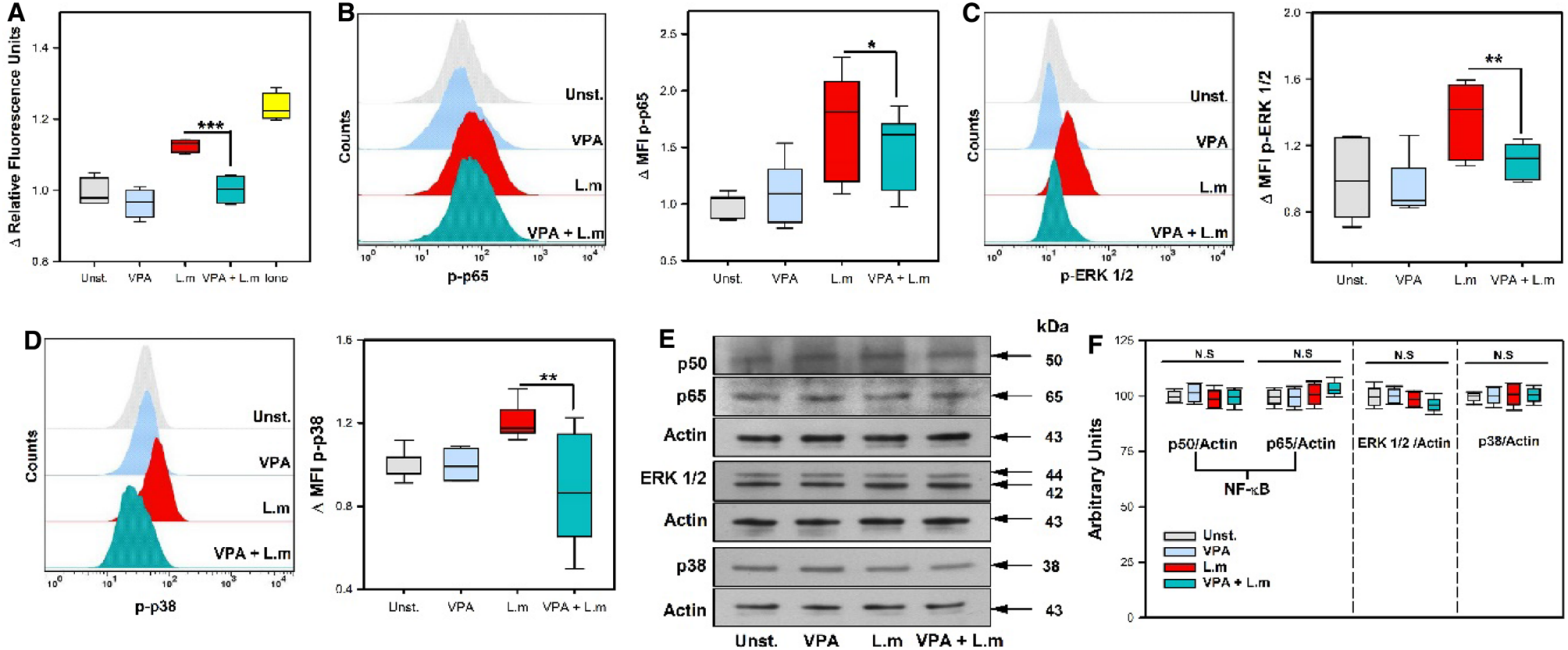
Valproic acid decreases calcium mobilization and phosphorylation of signaling proteins in *Listeria monocytogenes*-activated mast cells. (**A**) BMMC were pre-incubated with 2 mM VPA for 18 h and then stained with Fluo-4 AM. The baseline Relative Fluorescence Units (RFU) reading was recorded at 0 and 5 min in a fluorescence plate reader. Then, cells were stimulated with L.m or calcium ionomycin (iono), and RFU readings were recorded at 45 min. The panel shows the fold change in RFU with respect to the average value of the baseline RFU for each experimental group. (n = 4 per group; **p < 0.01; Kruskal–Wallis test). (**B**-**D**) BMMC were pre-incubated with 2 mM VPA for 18 h and then stimulated with L.m for different times to determine the phosphorylation of (**B**) NF-κB p65 at 30 min, (**C**) ERK 1/2 at 15 min and (**D**) p38 at 60 min by flow cytometry. The left panels show representative histograms of phosphorylated proteins. The right panels show the fold change of the median fluorescence intensity (MFI) with respect to the average value of unstimulated BMMC. (n = 6 per group; N.S = Not Significance, **p < 0.01, ***p < 0.001; Kruskal–Wallis test). (**E**–**F**) BMMC were pre-incubated with 2 mM VPA for 18 h and then stimulated with L.m for different times to determine the expression of (**E**) NF-κB p50 and p65 at 30 min, ERK 1/2 at 15 min and p38 at 60 min by Western-Blot. (**F**) Immunoblots were evaluated by densitometry. The data are expressed as arbitrary units, considering the average value of the expression of each protein in unstimulated cells as 100. (n = 4 per group; N.S = Not Significance; Kruskal–Wallis test). Uncropped western blot images are included in Supplementary figure S3.

Intracellular calcium mobilization is a crucial step in cell signaling associated with MC degranulation^31^. Then, we analyzed whether VPA interfered with Ca^2+^ mobilization induced by L.m. We noticed that 2 mM VPA treatment induced a significant decrease of intracellular Ca^2+^ mobilization in BMMC stimulated with L.m (Fig. 4A) (*p* < 0.001). Moreover, since VPA affected cytokine production in L.m-stimulated BMMC, we next evaluated if this drug could affect intracellular signaling molecules associated with this activation pathway. In this regard, it is known that the mitogen-activated protein kinase (MAPK) and NF-κB pathways are induced in macrophages infected with L.m, and that their activation is associated with cytokine synthesis^32–34^. In addition, recent work has demonstrated that L.m activates these two signaling pathways in MC^14^.

First, we noticed that 2 mM VPA significantly decreased NF-κB (p65) phosphorylation in L.m-stimulated BMMC, (*p* < 0.05) (Fig. 4B). Next, we evaluated the effect of VPA on the activation of MAPK signaling pathway. We found that VPA induced a significant decrease of p-ERK 1/2 (*p* < 0.01) (Fig. 4C) and p-p38 (*p* < 0.01) (Fig. 4D) in BMMC stimulated with L.m. As previous studies discovered that butyric acid, also a short-chain fatty acid with HDACi properties, inhibited the synthesis of key cell signaling molecules in MC^35^, we evaluated whether 2 mM VPA could inhibit the expression of p38, ERK 1/2 and NF-κB (p50 and p65) proteins in MC. Western-blot analysis revealed that VPA did not affect the expression of these polypeptides (Fig. 4 E,F; Fig. S3A-C). Therefore, our results indicate that VPA affects intracellular Ca^2+^ mobilization, MAPK and NF-κB activation in L.m-stimulated MC.

Altogether, these results show that VPA interferes with the activation of key cell signaling molecules, dampening MC activation in response to L.m infection.

## Discussion

Mast cells (MC) are cells of the innate immune system that participate in the control of different infectious processes through the release of preformed and de novo synthesized proinflammatory mediators that regulate the recruitment of effector cells to the site of infection^3^. In fact, MC initiate the response against *Listeria monocytogenes* (L.m) by promoting the recruitment of neutrophils to the site of infection in an experimental model of murine listeriosis^9^. Moreover, L.m activates MC by triggering TLR2 and through cholesterol-dependent activation by listeriolysin O (LLO)^14,36^.

VPA modulates several biological functions of cells of the immune response, including the activation of MC mediated by FcεRI crosslinking^25^. In this study we found evidence that VPA modulates MC response against L.m by affecting some of the activation mechanisms triggered by this pathogen.

VPA affected MC degranulation in response to L.m in a similar way to that observed in IgE/Ag-activated MC^25^ or NK cells in response to tumor cells^37^. Of note, other histone deacetylase inhibitors (HDACi) including trichostatin A (TSA), MGCD0103, and butyric acid also inhibit IgE/Ag-mediated MC degranulation^35,38,39^. One proposed mechanism for this inhibitory effect in MC degranulation is the ability of HDACi to alter the expression and activation of key signaling molecules^25,35^. MC degranulation depends on intracellular Ca^2+^ mobilization^31,40^. We noticed that VPA was able to interfere with MC intracellular Ca^2+^ rise induced by L.m. This ability of VPA to interfere with intracellular Ca^2+^ mobilization has been described in immune cells, like B lymphocytes^41^. However, the molecular mechanism implied is not fully understood. In MC, Ca^2+^ mobilization relies in the inositol-1,4,5-trisphosphate induced release of stored Ca^2+^ in the endoplasmic reticulum, which leads to the activation of the cell membrane channels ORAI1 and TRPC1 allowing the influx of extracellular Ca^2+^^42^. Whether VPA interferes with one or both sources of intracellular Ca^2+^ in MC needs to be further analyzed, although in neurons VPA induced the blockade of Ca^2+^ channels and depletes intracellular inositol^43^.

In our study, VPA did not affect MC respiratory burst. This result contrasts with the response observed in macrophages, in which VPA interferes with ROS production by reducing the expression of the membrane-associated subunits (gp91^phox^/NOX2 and p22^phox^), the cytosolic subunits (p47^phox^, p40^phox^ and p67^phox^) and the regulatory subunit (Rac2) of NADPH oxidase after activation with *E. coli* or *S. aureus*^24^. This difference could also be associated with variations in the mechanisms that regulate and/or activate NADPH oxidase, or with the different isoforms of NADPH oxidase present in these cells^44,45^.

On the other hand, VPA significantly reduced the cytokine production induced by L.m. This ability of VPA to regulate cytokine production has been observed in IgE/Ag-activated MC treated with different HDACi, including VPA^25^, butyric acid^35,46^ and TSA^39^. VPA also inhibits cytokine production in other immune cells, such as NK cells^23^, monocytes, macrophages and T cells, by affecting signaling pathways that are required for their production^19^, suggesting that VPA could target key cell signaling molecules that mediate MC activation during L.m infection.

To discern whether VPA affected receptors on MC involved in recognition of L.m^14,28^ we evaluated its effect on MC TLR2. We did not observe that treatment of BMMC with VPA affected cell surface expression of TLR2. This suggests that VPA differentially modulates the expression of activation receptors depending on the cell type. Previous studies showed a negative effect of VPA on the expression of TLR2, and other TLRs in macrophages^24^. Furthermore, the effect of VPA on the downregulation of activation receptors has also been reported in other immune cells including NK cells^47,48^ and MC, where VPA affected c-KIT and FcεRI^25^.

To test whether the ability of TLR2 to activate MC was affected by VPA, we evaluated the effect of VPA on activation with the classic TLR2 agonists *S. aureus* Peptidoglycan (PGN) and Pam3CSK4 (Pam). VPA decrease MC cytokine production by these TLR2 ligands, in similar way to what is observed in macrophages stimulated with Pam^22^. In addition, we observed that VPA inhibited MC activation with LLO, which activates MC in a TLR2-independent manner^14,30^ through cholesterol dependent activation^36^ mediated by ERK1/2 phosphorylation^49^. These observations indicate that VPA does not affect TLR2 expression in MC and suggest that this compound compromises L.m-mediated MC activation by modulating intracellular activation mechanisms involved in the MC response to this pathogen.

MAPK and NF-κB pathways are involved in cytokine synthesis by L.m-infected macrophages^32–34^ and are also induced in L.m-activated MC^14^. Our results indicated that VPA treatment decreased MC ERK 1/2, p38 and NF-κB phosphorylation during L.m infection. These results agree with observations that butyric acid and Trichostatin A (TSA) decreases the phosphorylation of ERK 1/2, p38 and NF-κB in IgE-activated MC^39,46^, an effect also associated with diminished production of IL-6 and TNF^46^. In addition, other studies have shown that VPA decreases p38 phosphorylation in macrophages^50^ and decreases p38 and NF-κB phosphorylation NK cells activated with IL-12 and IL-18^23^. Therefore, these findings suggest that VPA modulates activation MAPK and NF-κB in MC. Notably, previous reports have demonstrated that selective inhibition of p38, but not of ERK 1/2 diminishes the production of IL-10, IL-5 and IL-13 in LPS-stimulated MC^51^. Whether VPA interferes with the activation of other transcription factors in MC needs to be further analyzed, although in neurons VPA induced an altered activation of AP-1^43^.

Although VPA modulates activation of key molecules of cell signaling^52^, the mechanisms by which this drug affects cell signaling in immune cells are not fully understood. VPA is well known for their ability to function as an HDACi, which increases histone acetylation leading to modifications in gene expression^53^. Indeed, VPA suppresses NKG2D receptor expression in NK cells through HDAC3 inhibition, and this promotes an increase in histone H3 and H4 acetylation. Interestingly, VPA also decreases phosphorylation of STAT3, a transcription factor required for NKG2D expression^48^. It might be possible that, through its capacity to function as HDACi, VPA could promote the expression of molecules that inhibit specific signaling pathways. This has been observed with the HDACi TSA, which attenuates IgE/Ag-mediated MC activation by increasing the expression of the NF-κB inhibitor, I-κBα^39^. It is also possible that VPA increases the expression and activation of protein phosphatases as shown in dermal and epithelial cells^54,55^. Finally, it could be speculated that VPA inhibits the enzymatic activity of some protein kinases by binding to their catalytic or allosteric site, as suggested by modelling and structural studies of HDAC and VPA interactions^56^.

When considering the potential therapeutic effect of VPA a dual effect should be considered. In one hand, an increased susceptibility to infectious diseases as is observed in patients with bipolar disorder, where VPA treatment increased risk of respiratory infections^21^. Therefore, the possibility that treatment with VPA could increase the susceptibility of patients to infection by opportunistic pathogens should be considered, mainly in high-risk groups^57^.

**Figure 5:**
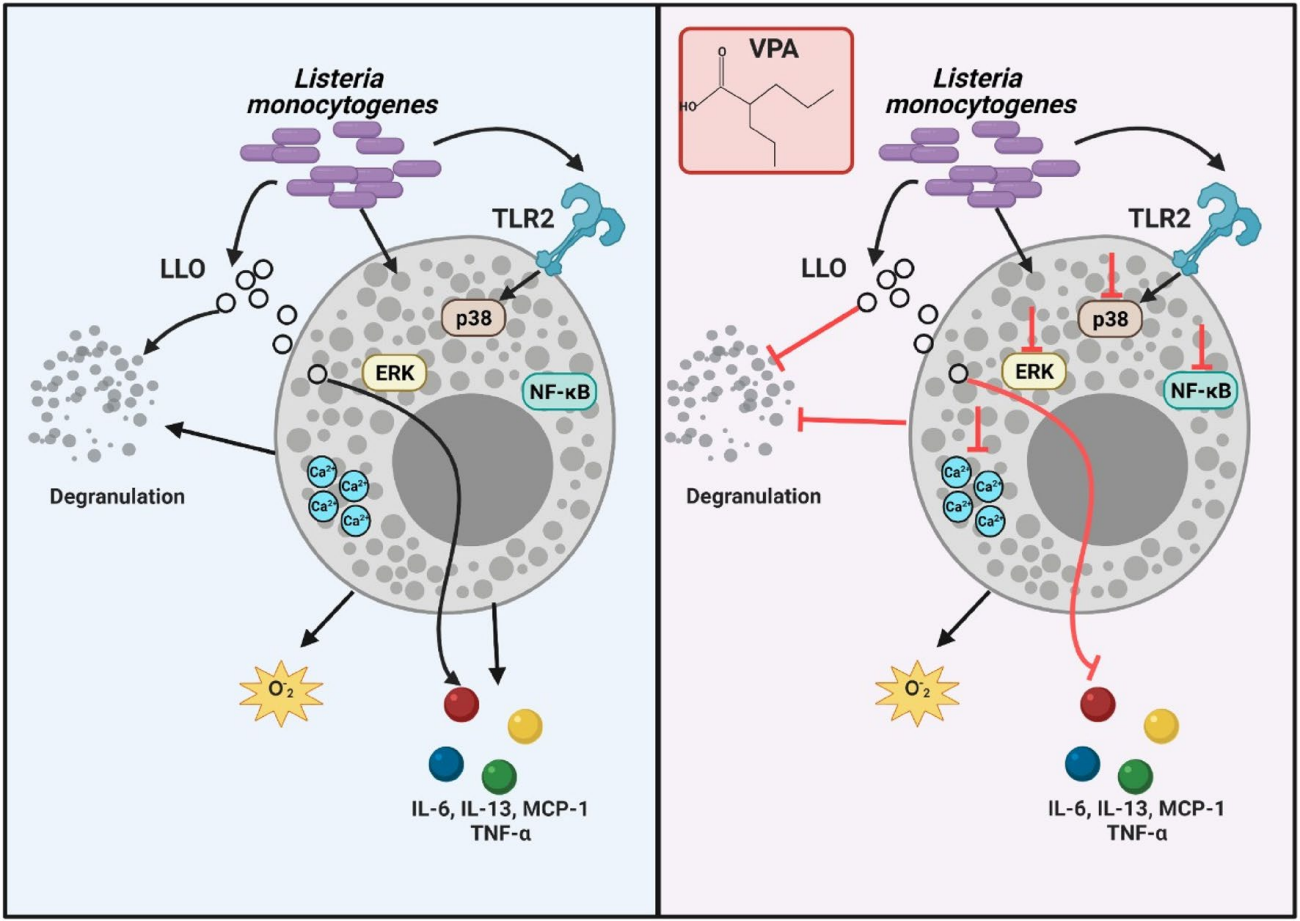
Valproic acid affects *Listeria monocytogenes*-mediated mast cell activation. Mast cells (MC) degranulate, produce superoxide anion (O^-^_2_) and secrete cytokines such as IL-6, IL-13, CCL2, and TNF in response *to Listeria monocytogenes* (L.m) infection. In addition, this pathogen induces MC intracellular Ca^2+^ mobilization and the activation of NF-κB (p65) and MAPK, ERK 1/2 and p38, the latter being dependent on TLR2-mediated signaling. In addition, Listeriolysin O (LLO) secreted by L.m induces MC degranulation and cytokine production (left panel). Pre-incubation of MC with valproic acid (VPA) affects MC degranulation, cytokine synthesis, intracellular Ca^2+^ mobilization and NF-κB, ERK 1/2 and p38 phosphorylation in response to L.m, as well as MC degranulation and cytokine production in response to LLO. Noteworthy, in this model VPA does not affect O^-^_2_ production, cell surface TLR2 expression (right panel). This figure was created with BioRender.com.

On the other hand, the drug repurposing potential of VPA as an anti-inflammatory agent should also be highlighted. In fact, the results of our study reinforce the role of VPA as a suppressor of MC activation, and this could be used in future studies to investigate its potential therapeutic effect in type I hypersensitivity reactions, mastocytosis and other infectious diseases characterized by MC hyperactivity^5,58–60^. In conclusion, our study demonstrates that VPA has a strong impact on the MC response to L.m infection by impairing the activation of key cell signaling pathways (Fig. 5).

## Methods

### Bacterial culture

*Listeria monocytogenes* (L.m) strain 1778 + H 1b (ATCC 43,249, Manassas, VA, USA) was grown in brain heart infusion broth (BHI, BD-Difco, USA) for 18 h at 37 °C with constant shaking at 112 xg. Bacterial cultures were washed with Hanks balanced saline solution (HBSS) (Life Technologies, USA) and bacterial pellets resuspended in RPMI-1640 Glutamax (Life Technologies, USA) supplemented with 40% fetal bovine serum (FBS) (Life Technologies, USA) and frozen at − 70 °C until use. Aliquots of L.m were serially diluted and plated in BHI agar at 37 °C for 18–24 h. Bacterial numbers were determined by counting colony-forming units (CFU).

### Mast cell culture

Bone marrow cells were obtained from femurs and tibias of 6–8-week-old C57BL/6 female and male mice. Cells were maintained in RPMI-1640 supplemented with 10% FBS, 5 μM β-mercaptoethanol (Life Technologies, USA) and 2% antibiotic and antimycotic (Sigma, USA) (complete RPMI 1640 medium), plus 10 ng/mL of murine recombinant IL-3 (Peprotech, USA) and 10 ng/mL of murine recombinant stem cell factor (SCF) (Peprotech, USA). Non-adherent cells were transferred to fresh culture medium twice a week for 6 − 9 weeks. The purity of bone marrow-derived mast cells (BMMC) was ≥ 90% as assessed by flow cytometry after staining of CD117 (clone: 2B8, BioLegend, USA; 0.25 μg/mL) and FcεRI (clone: MAR R-1, BioLegend, USA; 0.16 μg/mL).

### Ethical approval

All experiments followed institutional biosecurity and safety procedures. All methods were carried out in accordance to Mexican relevant guidelines and regulations. All animal procedures were reviewed and approved by the Research Ethics Committee of the ENCB, IPN (ZOO-017-2019), and the study complied with ARRIVE guidelines (Animal Research: Reporting of In Vivo Experiments).

### Valproic acid

An injectable solution of sodium valproic acid (Depakene®) was obtained from Hospira Inc. (Pfizer; Kansas, USA).

### Degranulation assay

2.5 × 10^5^ BMMC were cultured in 0.25 mL of complete RPMI-1640 medium with or without VPA at 2 mM, a concentration that does not affect cellular viability^23,25^, for 18 h. Cells were washed with HBSS and stimulated with L.m at multiplicity of infection (MOI) of 1:100 for 90 min. Following stimulation, cells were washed and stained with fluorochrome conjugated anti-CD107a (clone: 1D4B BioLegend, USA; 0.25 μg/mL) and anti-FcεRI antibodies. CD107a expression was measured by flow cytometry.

To evaluate β-hexosaminidase release 2.5 × 10^5^ BMMC were cultured in 0.25 mL of complete medium with or without 2 mM VPA for 18 h. Cells were washed with phosphate buffered saline (PBS) 1X (Life Technologies, USA) and cultured in 0.25 mL of HEPES-Tyrode Buffer (HBT) (130 mM NaCl, 5.5 mM glucose, 2.7 mM KCl, 1.0 mM CaCl_2_ 2 H_2_O, 0.1% [w/v] Bovine Serum Albumin (BSA), 12 mM HEPES, 0.45 mM NaH2PO4 1H2O, pH 7.2) with L.m MOI 1:100 or recombinant Listeriolysin-O (LLO; RayBiotech, USA) at 1000 ng/mL for 90 min at 37 °C. The supernatants were then recovered, and the cell pellet was lysed with 200 μL of 0.2% Triton X-100 in HBT. Both supernatants and cell lysates were incubated with 4-methylumbelliferyl N-acetyl-β-D-glucosaminide (Sigma-Aldrich, USA; 1 mM in 200 mM Na Citrate Buffer pH 4.5) for 2 h at 37 °C. The enzyme reaction was stopped by the addition of 100 μL of 200 mM Tris base, pH 10.7. The samples were analyzed in a fluorescence plate reader (SpectraMax M, USA) using excitation 356 nm and emission 450 nm. The percentage of release of β-hexosaminidase is calculated using the formula:

$$\% Release = \left[ {\frac{supernatant}{{\left( {supernatant + cell\ pellet} \right)}}} \right] x 100$$

The time of MC stimulation was selected based in a time-course assay, where the maximal release of β-hexosaminidase was detected at 90 min after incubation with L.m (Fig. S2A).

### Superoxide anion (O_2_^−^) production

2 × 10^5^ BMMC were cultured in 0.25 mL of RPMI-1640 without phenol red (Life Technologies, USA) supplemented with 10% FBS and 5 μM β-mercaptoethanol with or without VPA at 2 mM for 18 h. Then, the cells were washed with HBSS and stimulated with L.m MOI 100:1 for 30 min. In the last 15 min of incubation 25 μL of a solution of p-nitro blue tetrazolium (NBT, 1 mg/ml) (Sigma-Aldrich, USA) were added. Cells were washed and fixed with absolute methanol. The formazan precipitates were dissolved by adding 54 μL of 2 mM potassium hydroxide (KOH) and 46 μL of dimethyl sulfoxide (DMSO) (Sigma-Aldrich, USA). The samples were read at a wavelength of 620 nm in a plate reader (Multiskan EX, Thermo Scientific, USA).

The time of MC stimulation was selected based in a time-course assay, where the maximal ROS production was detected at 30 min after incubation with L.m (Fig. S2B).

### Cytokine quantification

2 × 10^5^ BMMC were cultured in 0.25 mL of complete medium with or without VPA at 2 mM for 18 h. Cells were washed with HBSS and activated with different stimuli: L.m MOI 100:1 or *Staphylococcus aureus* Peptidoglycan (PGN; Sigma-Aldrich, USA) at 10 μg/mL or Pam3CSK4 (Pam; InvivoGen, USA) at 10 μg/mL, or Recombinant Listeriolysin-O (LLO; RayBiotech, USA) at 1000 ng/mL for 24 h. Supernatants were collected for the detection of TNF, IL-6, CCL2 (BioLegend, San Diego, CA., USA) and IL-13 (eBioscience, USA) by ELISA according to manufacturer’s instructions.

### Toll-Like Receptor 2 expression in mast cell surface

2 × 10^5^ BMMC were cultured in 0.25 mL of complete medium with or without VPA at 2 mM for 18 h. Then, cells were washed with HBSS, Fc-γ receptors blocked with anti-CD16/32 (Mouse BD Fc Block™, clone: 2.4G2. BD-Biosciences, USA) and subsequently, incubated with anti-Toll-Like Receptor 2 (TLR2)-biotin (clone: 6C2; 1 μg/0.1 mL) or isotype controls (clone: eB149/10H5; Rat IgG2b, κ-biotin; 1 μg/0.1 mL, all from eBioscience, USA) for 1 h on ice. Finally, MC were stained with streptavidin-APC (BD-Bioscience, USA; 0.02 μg/mL) for 30 min on ice and protected from light. Staining was measured by flow cytometry.

### Calcium mobilization assay

2.5 × 10^5^ BMMC were cultured in 0.25 mL of complete medium with or without 2 mM VPA for 18 h. Cells were washed twice with HBT and stained with Fluo-4 AM (BD Pharmingen, USA) at 5 μM for 45 min at 37 °C. Subsequently, cells were washed twice with HBT and plated in a 96-well plate with 250 μL of HBT. Afterwards, the basal fluorescence reading was recorded at 0 and 5 min in a fluorescence plate reader at 37 °C, using excitation 490 nm and emission 516 nm. Then, HBT or L.m (MOI 100:1) resuspended in HBT was added, and fluorescence readings were recorded at 45 min. As a positive control, Ionomycin (Sigma-Aldrich, USA) was used at 20 μM.

The time of MC stimulation was selected based in a time-course assay, where the maximal intracellular Ca^2+^ mobilization was detected at 45 min after incubation with L.m (Fig. S2C).

### Evaluation of phosphorylated proteins

2 × 10^5^ BMMC were cultured in 0.25 mL of complete medium with or without VPA at 2 mM for 18 h, and then stimulated with L.m MOI 100:1 for 15 min to evaluate phosphorylated extracellular signal-regulated kinase 1/2 (p-ERK 1/2), 30 min for p-p65 and 60 min for p-p38, based on time-course assays previously reported^14^. Later, the cells were fixed with 250 μL of Fixation buffer (BD-Bioscience, USA) for 10 min/37 °C. Subsequently, cells were washed with 1 mL of Stain Buffer (BD-Bioscience, USA) and permeabilized with 1 mL of 0.5 × Perm buffer IV (BD-Bioscience, USA) for 15 min protected from light. Permeabilized cells were washed with 1 mL of Stain Buffer and Fc-γ receptors blocked with anti-CD16/32 (Mouse BD Fc Block™, clone: 2.4G2. BD-Biosciences, USA). Cells were stained with antibodies to p-ERK 1/2-PE (clone: 20A; 4 μL per tube), p-p65-PE (clone: K10- 895.12.50; 4 μL per tube), p-p38-PE (Clone: 36/p38; 4 μL per tube) or isotype controls (Clone: MOPC-21; Mouse IgG1, κ-PE or Clone: MCP-11; Mouse IgG2b, κ-PE; all from BD-Biosciences, USA) for 60 min protected from light. Finally, the cells were washed and resuspended in 0.15 mL of Stain Buffer and analyzed by flow cytometry.

### Western blot

3 × 10^5^ BMMC were cultured in 0.3 mL of complete medium with or without VPA at 2 mM for 18 h, and then stimulated with L.m MOI 100:1 for 15 min to evaluate ERK 1/2, 30 min for NF-κB p50 and p-65, and 60 min for p38. Then, the cells were collected by centrifugation and lysed in 300 μL of 1X Laemmli Buffer (Sigma-Aldrich, USA) supplemented with 4 mM sodium ortho-vanadate and 0.28 M β-mercaptoethanol. Proteins were resolved in 10% (p50) or 12% (ERK1/2 and p38) SDS-PAGE gels and transferred to polyvinylidene difluoride (PVDF) membranes, that, were then blocked with 5% low-fat milk (Svelty; Nestlé) and incubated overnight in TBS-T-buffer (25 mM Tris–HCl, 0.9% NaCl, 0.1% Tween 20) in the presence of primary antibodies at the following dilutions: ERK1/2, 1:10,000 (Cat. No. 9102; Cell Signaling, USA); p50, 1:1,000 (Cat. No. sc-1192); p65, 1:1,000 (Cat. No. sc-8008) p38, 1:1000 (Cat. No. sc-6187); β-actin, 1:10,000 (Cat. No. sc-81178); all from Santa Cruz Biotechnology, USA. After incubation with primary antibodies, membranes were washed three times with TBS-T-buffer before being incubated with the respective secondary antibodies: anti-mouse, 1:15,000 (Cat. No. 115–035-003; Jackson ImmunoResearch, USA); anti-goat, 1;15,000 (Cat. No. sc-2354, Santa Cruz Biotechnology, USA) or anti-rabbit. 1:15,000 (Cat. No. 111–035-003, Jackson ImmunoResearch, USA). Membranes were washed three times to remove the secondary antibodies, and chemiluminescent HRP substrate (Millipore-Sigma, USA) was used for the detection of protein bands. To obtain WB images, a piece of medical X-ray Blue/MXB film (Carestream Health Inc., USA) was placed on the zone of the expected molecular weight of each protein, determined by the pre-stained molecular weight marker PageRuler Plus 10–250 kDa (ThermoFisher Scientific, USA). Distinct exposure times of the X-ray films were utilized in order to avoid film saturation and only non-saturated films were used for densitometric analysis. Films were developed utilizing the Carestream® Kodak autoradiography GBX developer and fixer solutions (Sigma, USA). Relative quantitation of immunoblots was performed by densitometry using a Mini Bis Pro Bio System (DNR Bio Imaging Systems, Israel), utilizing the Image Studio Software version 5.2.5 included with the equipment. Uncropped western blot images are included in Supplementary figure S3.

### Flow cytometry

All cell samples stained with fluorochrome-conjugated antibodies were acquired using FACSCalibur (BD Biosciences) and analyzed with FlowJo software version 7.1 (FlowJo, LLC, USA) (https://www.flowjo.com/solutions/flowjo/downloads/).

### Statistical analysis

All statistical analyses were performed with SigmaPlot software version 14.0, from Systat Software, Inc., San Jose California USA (www.systatsoftware.com). Data normality was assessed by Kolmogorov–Smirnov with Lilliefors correction. Data are shown as mean ± standard error of the mean (s.e.m) or median and range (as appropriate) of 4–6 independent experiments. For comparisons between two groups, Mann–Whitney rank sum test with Yates correction was used. For comparisons between more than two groups, Kruskal–Wallis test with Student–Newman–Keuls (SNK) post-hoc was used. For comparisons between two or more groups with two factors, two way-analysis of variance (ANOVA) with SNK post-hoc was used. For comparisons between two or more groups with two factors and repeated measures, two way-repeated measures-ANOVA (RM-ANOVA) with SNK post-hoc was used. A value of p < 0.05 was considered to be significant.

## Supplementary Information

Supplementary Information.

## Data Availability

All data generated or analyzed during this study are included in this published article (and its Supplementary Information files).
