# Supplementary material for: Valproic acid restricts mast cell activation by Listeria monocytogenes
Source: Sci Rep. 2022 Sep 20;12:15685. doi: 10.1038/s41598-022-20054-5 (PMC9489790; doi:10.1038/s41598-022-20054-5)
Supplement: Supplementary file 1 — Supplementary Information. [file 41598_2022_20054_MOESM1_ESM.pdf]

# Valproic acid restricts mast cell activation by *Listeria monocytogenes*

Rodolfo Soria-Castro[1], Yatsiri G. Meneses-Preza[1], Gloria M. Rodríguez-López[2], Alfredo Ibarra-Sánchez[3], Claudia González-Espinosa[3], Sonia M. Pérez-Tapia[1,4], Fabián Flores-Borja[5], Sergio Estrada-Parra[1], Alma D. Chávez-Blanco[6]†, Rommel Chacón-Salinas[1]†.

1. Departamento de Inmunología, Escuela Nacional de Ciencias Biológicas, Instituto Politécnico Nacional (ENCB-IPN). Mexico City, Mexico.
2. Departamento de Microbiología e Inmunología, Facultad de Medicina Veterinaria y Zootecnia, Universidad Nacional Autónoma de México. Mexico City, Mexico.
3. Departamento de Farmacobiología, Centro de Investigación y de Estudios Avanzados (Cinvestav), Unidad Sede Sur. Mexico City, Mexico.
4. Unidad de Desarrollo e Investigación en Bioprocesos (UDIBI), Escuela Nacional de Ciencias Biológicas, Instituto Politécnico Nacional (ENCB-IPN). Mexico City, Mexico.
5. Centre for Oral Immunobiology and Regenerative Medicine, Barts & The London School of Medicine and Dentistry, Queen Mary University of London, London, United Kingdom.
6. Subdirección de Investigación Básica, Instituto Nacional de Cancerología (INCan). Mexico City, Mexico.

† Correspondence:

Rommel Chacón-Salinas, Ph.D.

Departamento de Inmunología, Escuela Nacional de Ciencias Biológicas, Instituto Politécnico Nacional, Carpio y Plan de Ayala s/n Col. Santo Tomás, México City. C.P. 11340, México. Phone: +525557296300 ext. 62507

E-mail: rommelchacons@yahoo.com.mx

or

Alma D. Chávez-Blanco, Ph.D.

Subdirección de Investigación Básica, Instituto Nacional de Cancerología (INCan). Av. San Fernando No. 22. Col. Sección XVI. C.P. 14080, México City, México. Phone: +52 56280400 ext. 31065

E-mail: celular_alma@hotmail.com

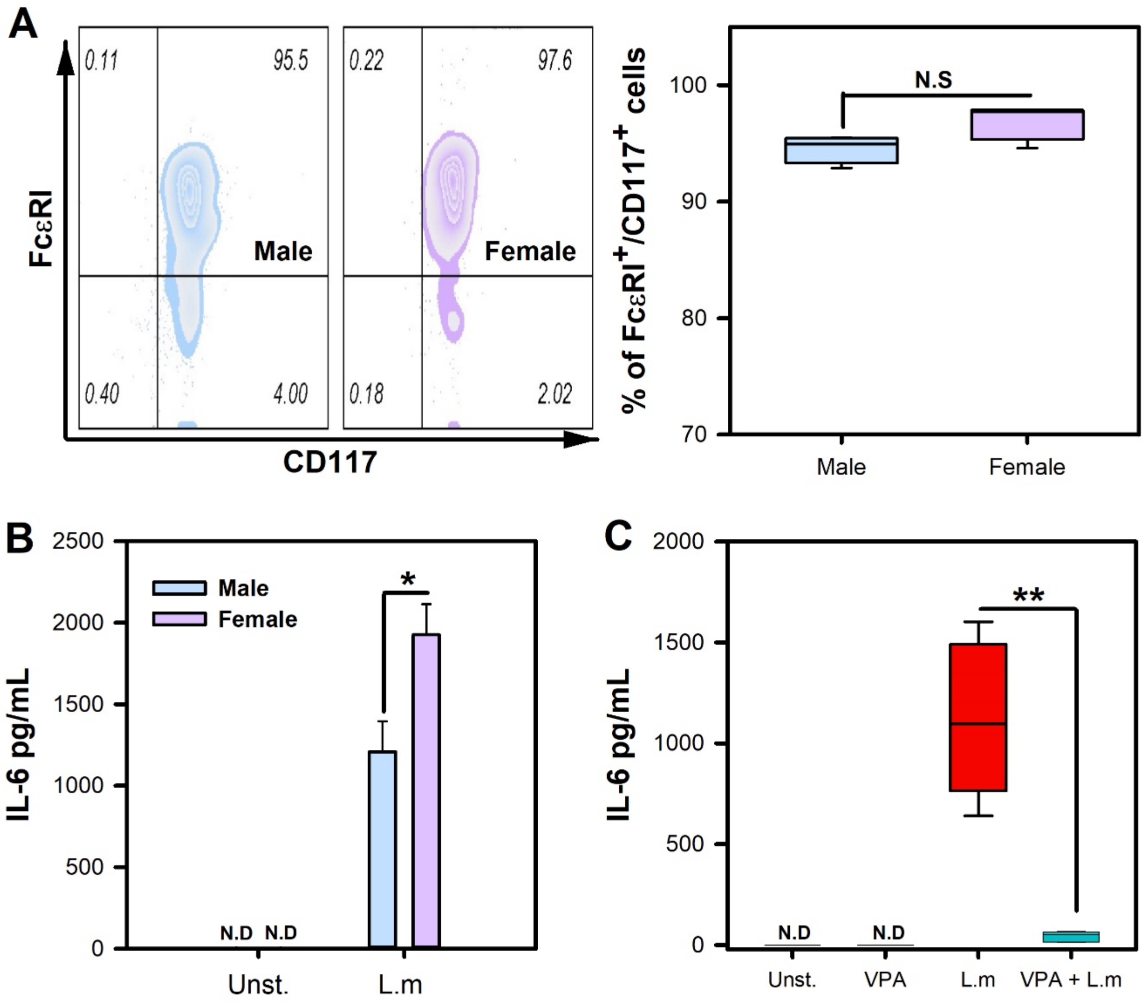


**Supplementary Figure S1. Valproic acid dampen male mast cell activation in response to *Listeria monocytogenes*. (A)** The left panel shows representative flow cytometry zebra plot of bone marrow-derived mast cells (BMMC) from male mice (blue) or female mice (pink). The right panel shows the percentage of FcεRI+/CD117+ cells. (n=4 per group; **N.S**= Not Significance; Mann-Whitney test). **(B)** Male or Female BMMC were stimulated with L.m for 24 h. IL-6 levels were evaluated in culture supernatants by ELISA. (n=4 per group; **N.D**= Not Detected; *p<0.05; two way-ANOVA). **(C)** Male BMMC were pre-incubated with 2 mM VPA

for 18 h and then stimulated with L.m for 24 h. IL-6 concentration was evaluated in culture supernatants by ELISA. (n=4 per group; **N.D**= Not Detected; **$p<0.01$; Kruskal-Wallis test).

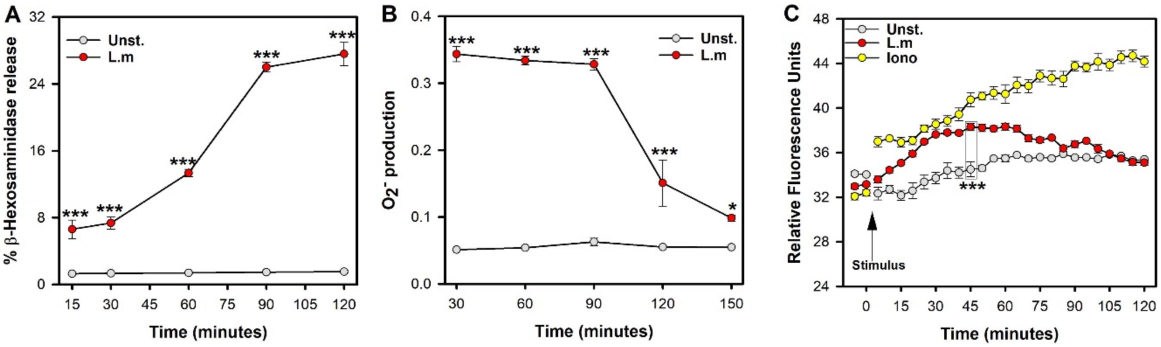


**Supplementary Figure S2. Time-course of mast cell degranulation, $O_2^-$ production and intracellular $Ca^{2+}$ mobilization induced by *Listeria monocytogenes*.** Bone marrow-derived mast cells (BMMC) were stimulated with *Listeria monocytogenes* (L.m) at different times. **(A)** Evaluation of degranulation by β-hexosaminidase release assay. (n=4 per group; ***p<0.001). Data are expressed as mean ± s.e.m; two way-ANOVA. **(B)** Evaluation of $O_2^-$ production through the NBT reduction assay. (n=4 per group; *p<0.05, ***p<0.001). Data are expressed as mean ± s.e.m; two way-ANOVA. **(C)** BMMC were stained with Fluo-4 AM. The baseline Relative Fluorescence Units (RFU) reading was recorded at 0 and 5 minutes. Then, cells were stimulated with L.m or calcium ionomycin (iono), and fluorescence readings were immediately recorded every 5 minutes for 2 h. (n=4 per group; ***p<0.001, Unst. vs L.m at 45 minutes). Data are expressed as mean ± s.e.m; two way-RM ANOVA.

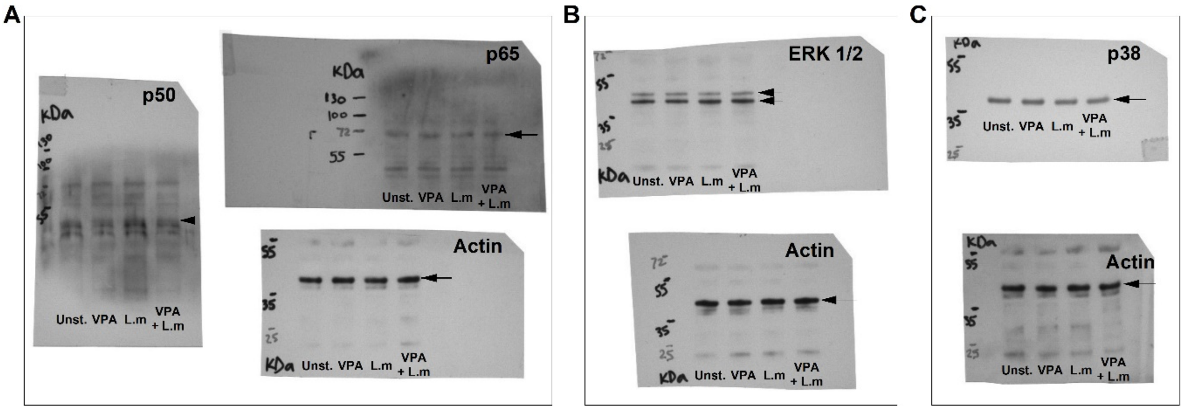


**Supplementary Figure S3. Uncropped western blot membrane images. (A)** X-ray films obtained from blot membranes for Fig 4E (NF-$\kappa$B: p50 and p65), **(B)** ERK 1/2 and **(C)** p38. Images were obtained as described in Methods section. Each panel shows the respective actin control and molecular size marker.
